# Needs‐driven talent and competency development for the next generation of regulatory scientists in Africa

**DOI:** 10.1111/bcp.15020

**Published:** 2021-08-24

**Authors:** Boitumelo Semete‐Makokotlela, Gugu N. Mahlangu, David Mukanga, Delese Mimi Darko, Peter Stonier, Luther Gwaza, Portia Nkambule, Precious Matsoso, Regine Lehnert, Bernd Rosenkranz, Goonaseelan (Colin) Pillai

**Affiliations:** ^1^ South African Health Products Regulatory Authority Pretoria Gauteng South Africa; ^2^ Medicines Control Authority of Zimbabwe Harare Zimbabwe; ^3^ Bill & Melinda Gates Foundation Seattle USA; ^4^ Food and Drugs Authority Accra Ghana; ^5^ Pharmaceutical Medicine Department, Institute of Pharmaceutical Sciences, Faculty of Health Sciences and Medicine Kings College London UK; ^6^ World Health Organization Geneva Switzerland; ^7^ Department of Pharmacy & Pharmacology, Faculty of Sciences University of the Witwatersrand South Africa; ^8^ GHPP‐PharmTrain project Federal Institute for Drugs and Medical Devices (BfArM) Germany; ^9^ Fundisa African Academy of Medicines Development Cape Town South Africa; ^10^ Division of Clinical Pharmacology, Faculty of Medicine and Health Sciences Stellenbosch University Cape Town South Africa; ^11^ Division of Clinical Pharmacology University of Cape Town South Africa; ^12^ CP+ Associates GmbH Basel Switzerland; ^13^ Pharmacometrics Africa NPC Cape Town South Africa

**Keywords:** Africa, capacity development, careers, public health, regulatory sciences

## Abstract

Capacity building programmes for African regulators should link education, training and research with career development in an approach that combines an academic base and experiential learning aligned within a competency framework. A regulatory ecosystem that engages with a broad range of stakeholders will mean that expertise in the ever‐expanding field of regulatory science filters into teaching and research in a symbiotic way. In this way capacity development interventions will be a collaborative approach between the learning context (academic and training institutions) and the performance context (regulatory agencies and industry), which will ultimately best serve the patients. Monitoring and evaluation of capacity development interventions will be essential to show value of investments and ultimately guide continued funding and sustainability.

This paper reviews the skills and human capacity gaps, reports on regulatory assessment pathways used in Ghana, South Africa and Zimbabwe and outlines a staged tactical approach for Africa that builds on previous efforts to strengthen African regulatory ecosystems.

## 1 INTRODUCTION

There is a critical skills gap on the African continent in regulatory sciences, and an acknowledged need to develop a long‐term strategy for training and professional development of African regulatory personnel.^1^, ^2^, ^3^, ^4^ Due to multiple factors including the low presence of research‐based pharmaceutical industries on the African continent, the available clinical pharmacology and regulatory sciences skills and expertise are extremely underutilized, often resulting in the flight of this talent to other parts of the world. The increase in new biotechnological products and advances in medical device technology as well as the evolving digital health landscape have challenged regulators across the world to stay updated with the development, manufacture and the use of these products in clinical settings.^5^ In Africa, regulators contend with the new challenges while still remaining *au fait* with regulations required for a generics‐dominated health care setting. Addressing these challenges requires an understanding of the regulatory environment to ensure effective and responsive regulation avoiding under‐regulation as well as over‐regulation. As resolved at the 67th World Health Assembly, effective regulatory systems are an essential component of health system strengthening, and inefficient regulatory systems can be a barrier to access safe, effective and quality medical products.^6^ In addition, effective regulation contributes towards promotion of public health.

Fortunately, the continent is home to several regulatory agencies that drive a science‐based agenda. This can be expanded and agencies can be strengthened if their current human capacity could be further developed. Several international initiatives have been established to define competency frameworks for medicines development and to harmonize education and training. The World Health Organization's (WHO) draft competency framework for regulators,^7^ education and training curricula in pharmaceutical medicine^8^ and the African Medicines Regulatory Harmonization initiative^9^ could be used to guide development of African training programmes for regulators.

In June 2020, the South African Health Products Regulatory Authority (SAHPRA) hosted a virtual meeting to share experiences, inspire local regulators, build networks and develop a programme of action on the development of capacity and capabilities in African regulatory agencies. The meeting comprised 40 senior level participants from African and non‐African regulatory authorities, the WHO, African Union Development Agency (New Partnership for Africa's Development) as well as local and international academics. Following a few short contextual presentations by regulatory experts and academics, a longer facilitated open discussion ensued. A full report on the meeting, including summaries of the presentations is available on the SAHPRA website.^2^ The outputs from that meeting, and a review of the regulatory assessment pathways used in Africa were combined with a targeted literature search to contextualize the authors' collective experiences on the need for education and training in regulatory sciences in Africa. The purpose of this paper is to invite comment, critique and offers of collaboration.

## 2 THE REGULATORY ASSESSMENT PATHWAYS USED IN AFRICA

Most national regulatory agencies in Africa have regulatory guidances and procedures in place for review of medicinal products.^1^, ^3^, ^10^ The regulatory assessment pathways involve **full independent reviews**, **work‐sharing or joint reviews, reliance,** or **recognition** of outcomes conducted by other agencies based on well‐defined regulatory processes. The proportion of dossiers handled via the first 3 assessment pathways is shown for Ghana, South Africa and Zimbabwe in Table 1, and could be considered illustrative for most African agencies. Table 1 shows that in South Africa, the transition from the Medicines Control Council to the new agency, SAHPRA has resulted in an increase in the use of reliance procedures compared to the past,^11^ and also provides data on a special project to clear an inherited backlog of dossiers.^12^

**TABLE 1:** Regulatory assessment pathways used by Ghana, South Africa and Zimbabwe during January to December 2020

Country	Full review^1^	Collaboration^2^	Reliance^3^
Ghana *n* = 958	922 (96.2%)	11 (1.1%)	25 (2.6)%
South Africa—Current dossiers *n* = 359	196 (55.1%)	14 (3.9%)	149 (41%)
South Africa—Backlog project^4^ *n* = 178	92 (51.7%)	0 (0%)	86 (48.3%)
Zimbabwe *n* = 191	148 (77.5%)	21 (10.9%)	22 (11.6%)

^**1**^ Full review: a complete independent review by the agency of the pharmaceutical quality via chemistry, manufacture and controls, assessment of safety and efficacy of preclinical and clinical data or bioequivalence assessment for generics.

^**2**^ Collaboration: involves a complete review but uses information or work sharing among participating organizations—either within regional collaborating groups such as ZAZIBONA in Africa or with selected international partners.

^3^ Reliance: is employed for products already approved by other stringent regulatory agencies and involves either a verification review process to validate that the product conforms to the previously authorized specifications or an abridged evaluation that takes into consideration local factors and environment.

^4^ SAHPRA inherited medical products dossiers from its predecessor, the Medicines Control Council that dates back to 1992, which is being cleared via a detailed separate strategy from the *business as usual* (current) dossiers.

The **full independent review** pathway involves a complete review by the agency of the pharmaceutical quality via chemistry, manufacture and controls as well as safety and efficacy assessment of preclinical and clinical data.

**Work‐sharing** or **joint reviews** involves the participation of 2 or more regulatory agencies such as the Southern African Development Community (SADC)^13^ and the East African Community.^14^ The global access mechanisms such as EUM4All^15^ or the Swissmedic Marketing Authorization for Global Health Products^16^ are hybrid examples that combine joint reviews and information sharing (reliance).

**Reliance** involves either a verification or an abridged review^17^, ^18^ and takes into account, either partly or fully, the assessment done by other agencies. The collaborative procedures for WHO‐prequalified products or products approved by stringent regulatory authorities are examples of established reliance or recognition mechanisms as applicable to individual participating authorities.^19^, ^20^

**Recognition** is the acceptance of the regulatory decision of another regulator or trusted institution, and could be unilateral or mutual. Mutual recognition is based on treaties or equivalent agreements, providing maximal benefits but partial loss of sovereignty concerning decision‐making.^21^ To our knowledge, recognition, whether unilateral or mutual, is less practiced on the African continent.

It is worth noting that challenges, often due to the lack of or limited expertise to conduct preclinical and clinical assessment, are mostly encountered with the full independent review or reliance on agencies for which context or populations are different. This is of particular concern when dealing with medicinal products that might be affected by differences due to intrinsic e.g. genetic makeup or extrinsic factors as defined in the ICH‐E5 guidance.^22^ This has implications when sponsors extrapolate data from clinical trials where African patients are underrepresented. Regulatory agencies on the continent are presented with the daunting task of assessing benefit–risk based on incomplete safety and efficacy data in the African population.

## 3 AFRICAN REGULATORY AGENCIES HAVE A SKILLS AND HUMAN CAPACITY GAP

Most of the African regulatory agencies have persistent human resource gaps leading to perennial backlogs, lengthy review timelines^11^ and challenges in meeting the best practices defined by stringent regulatory agencies across all areas of regulatory sciences.^3^ The human resource gaps are multifaceted—ranging from inadequate staffing numbers (too much work for too few staff), job descriptions that do not adequately specify the competencies required for the positions,^3^ inadequate human resource supply from academic programmes, low diversity of scientific expertise, and the inability of the health agencies to attract and retain qualified and experienced staff.^2^ Some of the issues are more prominent in some agencies than others; for example, in some countries, retention may be high but the required skill‐sets are not available or the staffing levels are not aligned with the workload demands.

### 3.1 Staffing numbers and vacancies

There are low staff numbers relative to the high workload in African settings compared to well‐resourced agencies outside Africa. This is the starting point of the problem. In a report on human resource capacity in regulatory agencies in 11 countries in the SADC in 2010, the number of regulatory evaluators ranged from zero filled posts in most of the countries to (only) 36 of 63 filled posts in South Africa. The other countries reported between 1 and 8 positions for this key role of regulatory evaluator.^3^ Based on the presentations by the heads of the national regulatory agencies at the June 2020 regulatory capacity strengthening meeting, it was further confirmed that these statistics have not changed much over the past decade—there is still a large human resource gap in regulatory agencies across the continent.^2^

### 3.2 Staff retention

Most agencies hire recent graduates who undergo in‐house or on‐the‐job training,^23^ often taking years to reach a level at which they start to be productive in terms of quality and quantity of outputs. However, due to the demand for skilled people especially those with experience gained at a regulatory agency, there is a struggle to retain staff with agencies finding themselves in a perpetual circle of training young graduates who move to the private sector just when they are beginning to become productive regulators. In many countries, the health authorities' financial rewards are not comparable with private sector, resulting in high rates of staff attrition and vacancies.^2^

### 3.3 Competency requirements

A contextual competency driven approach will help regulatory agencies not only to clearly define their human resource needs in terms of skills, knowledge and behaviours but also to monitor effectiveness of any capacity development interventions. Without clearly defined competency requirements that are aligned with the agency's needs and strategic plans and consistent with international best practices, African regulatory agencies lack the frameworks and data to support evidence‐based human resource development.

This has led to application backlogs at many agencies^11^ as well as use of external evaluators,^10^ usually academics, who have to take on this extra regulatory review work along with an already heavy teaching, research and health care service provision workload, which leaves no time for skills transfer or mentoring of regulatory staff.^2^

The skills gaps are larger among agencies with limited budgets since most depend on their Ministry of Health and Ministry of Finance for funding and are often under‐resourced given competing budget priorities. Some agencies such as the Ghana Food and Drugs Authority and the Tanzania Medicines and Medical Devices Authority, the 2 agencies in Africa that have attained WHO Maturity level 3,^7^ generate income internally and allocate a portion towards capacity strengthening activities. However, the majority need to raise funding for, or find partners to support specific training programmes and for attracting and retaining qualified and experienced staff. The size and research intensity of the pharmaceutical sector in the respective countries also plays a critical role in determining availability of the required skills sets.

**Why is it important to have the required skills sets within the regulatory agencies?**

- Regulators play a central role in enabling access to medicines that are safe, efficacious and of quality on the continent as well as educating the communities on the implications and caveats associated with new medicines.
- Regulators provide oversight for the entire product lifecycle from development of medicines through their license to the maintenance of medicines on the market by evaluating if emerging safety data (pharmacovigilance) changes the risk–benefit profile.
- The evolving nature of modern pharmaceutical interventions (e.g. medical devices, companion diagnostics, personalized medicine and digital medicine) motivates for hiring from an expanded pool of core skills that includes medical, engineering and allied quantitative sciences in addition to the traditional focus on the pharmaceutical sciences.
- African traditional and herbal medicines have a long history of use in the prevention and treatment of human and animal diseases. Regulators will need to identify complementarity, mutual respect and partnership between modern and traditional medicine.
- The creation of a conducive research and development and clinical trials environment will attract international players and financial investments and expected knock‐on effects for improvements in capabilities, standards, practices and accountabilities.
- African patients are consistently under‐represented in global pivotal clinical trials as shown by an examination of trial registries such as clinicaltrials.gov. It is therefore often guesswork when one extrapolates study results for those interventions that are suspectable to the ICH‐defined intrinsic and extrinsic factors that impact treatment outcome.^22^ Regulatory science training must include an approach that calls for generating specific data when scientifically warranted.

## 4 BUILDING FROM EXISTING PROGRAMMES AND EXPERIENCES

Recognizing that even the well‐established international regulatory agencies continue to invest heavily in on‐going professional development of their own staff, any new programme developed for African regulators should build from the baseline that exists by accessing content from international, regional and local programmes that are already at a sophisticated level of development. Some of these programmes have been described in the literature.^8^, ^24^, ^25^ The need for capacity building in clinical research has been recognised in a guidance documented published by SAHPRA.^26^

Accredited programmes should be integrated, as appropriate, with innovative and relevant teaching and training approaches as recommended by several thinktank reports.^27^, ^28^, ^29^

When coupled with career pathways as illustrated in Table 2, this could be especially powerful. The model presented in Table 2 is an example that shows the skills progression in a discipline from foundation to expert level based on Dreyfus' 5‐stage model of adult skill acquisition.^30^ The model will need to be adjusted based on local agency specificities e.g., combining job titles and discipline specific expertise depending on institutional context.

**TABLE 2:** An illustrative model to link competency levels, accreditation and career paths

Competency level, focus of learning efforts or role description	Accreditation/certification requirements	Link to career path or job titles
Foundation level (level I) Applies prior knowledge and skills while learning regulatory frameworks, requirements, legislation, and processes	Number of core modules; number of hours of subject matter experience gained *on‐the‐job* and work outputs documented in a portfolio	Regulatory officer
Intermediate level (level II) Learns all technical aspects of regulatory tasks connected with specified areas of regulation	Number of core modules; number of hours of subject matter experience gained *on‐the‐job* and work outputs documented in a portfolio; postgraduate certificate or diploma qualification	Senior regulatory officer
Advanced level (level III) Transitions into using technical knowledge into regulatory strategy.	Number of core and supplementary modules; number of hours of subject matter experience gained *on‐the‐job* and work outputs documented in a portfolio; research project with peer‐reviewed publications; masters‐level or doctoral qualification	Regulatory expert
Expert level (level IV) Strategic regulatory leader who develops new approaches based on a sound understanding of regulatory requirements, opportunities, risks and alternatives.	Number of additional core and supplementary modules, number of hours of subject matter experience gained *on‐the‐job* and work outputs documented in a portfolio; research in the regulatory sciences documented with peer‐reviewed publications masters‐level or doctoral qualification	Senior regulatory expert

The competency‐driven programmes follow a professional qualification approach and might be assessed for a specified area of regulation e.g. clinical trials regulation. The number of competency levels may be reduced or expanded depending on the size of agencies. The accreditation requirements might be aligned with internal agency job profiles and the qualifications (diploma, masters or doctorate) offered by local academic institutions. Suitable academic degrees or work experience documented in a portfolio may replace attendance of modules as recognition of prior learning. Competency assessments may be waived if modules are completed within an accredited network of course providers.

The WHO offices in Africa recognized the need to simultaneously strengthen regulatory systems while building capacity, e.g. the joint review programme of the African Vaccines Regulatory Forum, has been critical in bridging capacity gaps since its inception in 2006. The WHO prequalification process allows for rotational fellowships, bimonthly joint assessment sessions and also offers assessor training events.^31^ The Zazibona collaborative medicines registration initiative^13^ has similarly supported building capability in medicine assessments.

A harmonization initiative for medical products regulatory systems in Africa was endorsed by African Heads of State and Government at the January 2016 AU Summit in Addis Ababa, Ethiopia.^9^ The New Partnership for Africa's Development‐led programmes included efforts to initiate the designation of Regional Centres of Regulatory Excellence (RCOREs) across the African continent with the aim to support a regulatory workforce that enhances human and institutional capacity and contributes to improved health care delivery, regulatory standards, and practices in Africa.^32^ As an implementation example, the Ghana Food and Drugs Authority was designated as an RCORE for Clinical Trials, Drug Registration and Pharmacovigilance.^32^ As an RCORE, the agency collaborates with the School of Public Health, University of Ghana, thereby entrenching academic recognition of its programmes.

The draft WHO Global Competency Framework aims to address the gap around global competencies for regulators across all the currently defined regulatory functions that is flexible and adaptable by agencies at different maturity levels to meet their current and future needs.^7^ The framework can inform evidence‐based human resource approaches from recruitment, education and training to professional/career pathways and is flexible to allow individual regulatory authorities to adopt it based on their needs and local context. The draft has been piloted in Botswana's and SADC's regional joint activities, and provides the opportunity to benchmark the current technical skill base of existing regulatory teams and help design career development pathways.

## 5 CONCEPTUAL FRAMEWORKS FOR AN AFRICAN REGULATORY EDUCATION AND TRAINING SYSTEM

The preferred approach would be an over‐arching structure, e.g. a competency framework along the lines of that set up by the WHO. This will allow for specifying different levels of proficiency for different roles as well as establishment of competency‐related benchmarking tools (see Table 2 for an illustrative example).

The home for such a competency framework might take the form of a Regional Institute (or Academy) of Regulatory Sciences in Africa with an inclusive membership. Inclusivity will allow for minimal compromise of the sovereignty of regulatory agencies in African member states and should ensure involvement of academia for postgraduate accreditation.

The concept of reliance as a mechanism to increase efficiency has been promoted in Africa,^17^ but implementation varies widely (Table 1). Regulatory reliance is defined by the WHO as “The act whereby the regulatory authority in one jurisdiction may take into account and give significant weight to—i.e. totally or partially rely upon—evaluations performed by another regulatory authority or trusted institution in reaching its own decision. The relying authority remains responsible and accountable for decisions taken, even when it relies on the decisions and information of others.^33^ Reliance is a mechanism that can reduce the human resources workload while still accounting for the specific competency requirements for regulation of innovative products in Africa.^17^ It would be prudent to reiterate here that national agencies will always retain responsibility for pharmacovigilance since this is pursued nationally.

## 6 IMPLEMENTATION TACTICS

An African education and training solution for regulators might be implemented by learning from others' experiences and involvement in international guideline development. Historically, many low‐ and middle‐income countries have not been involved in the actual decision making around the creation of guidelines and the building of regulatory (systems) guidelines.^2^ The iterative cycles involved in developing guidelines are a way to build capacity since expertise is cultivated when one is engaging with the thought processes of how to develop the standards that one is required to apply.

Short‐, medium‐ and long‐term tactics should be considered. Duplication will be avoided by incorporating programmes, content or lessons learnt from other African and international regulatory capacity strengthening initiatives.

The meeting hosted by SAHPRA suggested that the types of training envisaged here should be done as extramural postgraduate courses to be contextual, i.e. undertaken while *on‐the‐job* to allow experiential influences, coupling these with mentorship programmes that are guided towards complementing the context with real‐life examples and anecdotes during mentor–mentee discussions. This could gradually be complemented with transfer of training content into under‐graduate programmes to improve the relevance of the training.

In well‐resourced regulatory agency settings, instruments for training are tailored towards the role in the agency that the staff member plays, e.g. a programme for clinical assessors will be very different to that of a regulator who assesses quality aspects of a biological. In African settings, where the currently limited staff are expected to cover multiple roles, this level of specialization should be an aspirational goal contingent on practicalities.

Fees charged by regulatory agencies are not related to the true cost of the pharmaceutical regulatory process.^34^ The potential revenues generated by the African market, coupled with revenues from agency fees, could provide resources to finance efforts to build capacity and capability of regulators. While regulatory agencies in larger markets are able to charge higher fees that are channelled towards building staff capacity and capabilities, this might not be easily implementable in Africa.

Regarding on‐the‐job training, local and international regulators have often hired subject matter experts (e.g. clinical specialists) into their agencies. These new staff then undertook internal training courses to learn regulation and regulatory processes. Arguably, this is a more efficient process than training a technically competent regulator on pharmaceutical or clinical subject matter specificities.

### 6.1 Short‐term tactics (within 6 mo)

- Conduct a detailed gap analysis using multiple sources of information including published reports, feedback from agency staff, industry and other stakeholders of the African regulatory ecosystem.
- Strive for Africa‐centricity in terms of local needs, training content and faculty.
- Apply modern methods of vocational education and training, especially those that use technology to increase efficiency and flexibility for educating professionals who are in full‐time employment.
- Execute at least 1 training event that fills an urgent need and could also help to illustrate aspects of the longer‐term plan and test out how trainings link certification to career paths as illustrated in Table 2.

### 6.2 Medium‐term tactics (within 2 y)

- Establish a quality assurance process for courses and training centres and a monitoring and evaluation programme for all interventions using metrics/tools that have been established for assessing similar programmes.
- Set up a close working relationship between regulatory agencies and academia that could simultaneously provide local accreditation and higher qualifications to agency staff who acquire advanced skills to execute their roles. This will naturally entrench the system of regulatory reliance where agencies consider each other's assessments. Consider regular (e.g. bimonthly) formal (virtual) meetings between the key stakeholders.
- Commence advocacy programmes to attract staff from a more multidisciplinary background e.g. engineering and allied quantitative sciences, in addition to pharmacy and the clinical sciences that have traditionally attracted regulatory professionals.
- Implement additional, nonmonetary incentives such as an African research agenda for regulatory sciences to attract and retain young scientists in the field.

### 6.3 Long‐term tactics (3–5 y)

- Develop a long‐term strategic plan for strengthening African regulatory sciences and capabilities, including standard setting for competency domains, learning outcomes and syllabus, creating an enabling workplace to encourage training, mentoring and assessments. This should involve compiling an inventory of available education and training programmes/platforms that already operate in Africa.
- Seek out, align with and form collaborations with other global initiatives that have similar objectives.
- Identify compliant mechanisms to partner with the pharmaceutical industry for combined training of industry and agency regulatory scientists
- Conduct contextual research into the regulatory sciences by existing regulatory staff, complemented by capacity and skills development to fulfil this research role.

The authors remain committed to implementing on the tactics listed in this section and reiterate the call for potential collaborators to contact the corresponding author directly or via the Journal. This represents a major goal with publishing this manuscript.

## 7 MEASURING IMPACT AND ENSURING SUSTAINABILITY

Prospective monitoring and evaluation of programmes as described here should be undertaken. Logic frames should be established for the projects/programmes, including measures for input, reach, output, outcome and impact representing the increasing levels of change management envisaged. Accompanying evaluations of programmes, and particularly assessment of (long‐term) impact is not an easy exercise, but nevertheless is needed to show value of investments and will ultimately guide continued funding and help sustainability.

Since governments are implicitly involved, or are hosts to the regulatory agencies if housed in relevant Ministries of Health or Science and Technology, they have a dominant effect on creating an enabling environment for regulatory capacity development. Collaboration of different government departments seeking complementarity in the interests of their communities e.g. the ministries of science and technology, health, finance, and trade will create business environments that could attract multinational pharmaceutical companies.

## 8 CONCLUSIONS

Capacity building programmes for African regulators should link education, training and research with career development in an approach that combines an academic base and experiential learning aligned within a competency framework. A regulatory ecosystem that engages with a broad range of stakeholders will mean that expertise in the ever‐expanding field of regulatory science filters into teaching and research in a symbiotic way. In this way, capacity development interventions will be a collaborative approach between the learning context (academic and training institutions) and the performance context (regulatory agencies and industry), which will ultimately best serve the patients.

## COMPETING INTERESTS

The authors declare that the research was conducted in the absence of any commercial or financial relationships that could be construed as a potential conflict of interest.

## CONTRIBUTORS

Substantial contributions to the conception and design of the project: B.S.M., G.P., D.M., G.N.M., B.R., P.S. Wrote the first draft of the manuscript: G.P., B.S.M., B.R., P.S., D.M. Revised the first draft critically for important intellectual content: B.S.M., G.N.M., D.M., M.D., P.S., L.G., P.N., P.M., R.L., B.R., G.P. Reviewed and approved the final version of the manuscript: B.S.M., G.N.M., D.M., M.D., P.S., L.G., P.N., P.M., R.L., B.R., G.P.
